# Development and Validation of Multiplex Quantitative Real-Time PCR Assays for Simultaneous Detection and Differentiation of HTLV-1 and HTLV-2, Using Different PCR Platforms and Reagent Brands

**DOI:** 10.3389/fmicb.2022.831594

**Published:** 2022-03-15

**Authors:** Maria Gisele Gonçalves, Lucila Okuyama Fukasawa, Karoline Rodrigues Campos, Fábio Takenori Higa, Adele Caterino-de-Araujo

**Affiliations:** Centro de Imunologia, Instituto Adolfo Lutz, Coordenadoria de Controle de Doenças, Secretaria de Estado da Saúde de São Paulo, São Paulo, Brazil

**Keywords:** HTLV-1, HTLV-2, multiplex qPCR, *pol*, *tax*

## Abstract

Brazil currently has the highest number of individuals infected with human T-lymphotropic virus 1- and 2- (HTLV-1 and HTLV-2) globally. At present, neither molecular protocols nor commercial assays are available for HTLV-1/-2 diagnosis or validated by the Brazilian Ministry of Health regulatory agency (ANVISA). We developed and validated two in-house multiplex quantitative real-time PCR for HTLV-1/-2 (mqPCR_HTLV) assays, targeting the *pol* and *tax* genes, for the simultaneous identification of HTLV-1, HTLV-2, and the albumin reference gene. The robustness of the assays was evaluated on two platforms using seven commercial master mix formulations. The reactions employed double plasmids (pHTLV1-Alb and pHTLV2-Alb) for the standard curve’s construction and for expressing the detection limit of the assays. They were able to detect 10 and 10 copies of HTLV-1 and 10 and 70 copies of HTLV-2 for the *tax* and *pol* targets, respectively. High efficiency was obtained using both the platforms and all the reagents evaluated and were successfully reproduced by other analysts. DNA samples from HTLV-1/-2-infected and non-infected patients and from HIV/HTLV-coinfected patients were evaluated to determine the feasibility of their use in routine diagnosis. The mqPCR_HTLV (*pol* and *tax*) assays demonstrated an overall specificity of 100% and a sensitivity of 97.4% when testing samples from patients without HIV infection, and sensitivities of 77.1% (*pol*) and 74.6% (*tax*) in samples from HIV/HTLV-coinfected patients. In addition, they resolved the issue of HTLV western blotting (WB) indeterminate and WB-untyped results in 45.5 and 66.7% of cases, respectively. The developed mqPCR_HTLV (*pol* and *tax*) assays indicated their feasibility for efficient and reliable HTLV diagnosis in various core facility laboratories under different conditions and supplies.

## Introduction

Human T-lymphotropic virus 1 and 2 (HTLV-1 and HTLV-2) are endemic to Brazil and their distribution varies according to the ethnic background of individuals and geographic regions (CGIST/DDCI/SVS et al., 2020). Brazil has the highest number of HTLV-1/2-infected individuals in Latin America and worldwide (Gessain and Cassar, 2012; CGIST/DDCI/SVS et al., 2020). Although the majority of the individuals remain asymptomatic, many people living with HTLV may develop one or more of a range of related diseases (Haziot et al., 2019; Rosadas et al., 2021a). At least two diseases of high morbidity and mortality rates are associated with HTLV-1, including HTLV-1-associated myelopathy (HAM) and adult T-cell leukemia/lymphoma (ATL), while HTLV-2 is rarely associated with a disease (Paiva and Casseb, 2015; Rosadas et al., 2021a). Thus, it is important to perform confirmatory and discriminatory diagnosis of HTLV-1/2 infections. There is no curative treatment available for these infections, therefore preventive measures, which include correct diagnosis, are the only means to eliminate HTLV transmission (Rosadas et al., 2021b).

The laboratory diagnosis of HTLV-1 and -2 is performed on blood samples from patients using screening tests followed by confirmatory tests. The screening tests detect antibodies against HTLV-1/2 in plasma/serum samples; they have high sensitivities, and negative results exclude HTLV-1/2 infection (da Silva Brito et al., 2018; Caterino-de-Araujo et al., 2021). The most common screening tests are the particle agglutination (PA), enzyme immunoassays (EIA or ELISA), and chemiluminescence immunoassays (CLIA) (Okuma et al., 2020; Caterino-de-Araujo et al., 2021; Rosadas et al., 2021a). Confirmatory serologic tests identify antibodies specific for HTLV-1 and -2 antigens in plasma/serum samples, and the most common types of these tests are western blotting (WB) and the line immunoassay (LIA). Both these serologic confirmatory assays help in diagnosis, but in some cases, inconclusive results may occur (HTLV untyped and indeterminate), especially when samples obtained from patients with HTLV-2 infection and those from patients coinfected with HIV, and/or HBV, and/or HCV (Campos et al., 2017a,b, 2020; Caterino-de-Araujo et al., 2021) are tested. LIA has a greater accuracy in comparison to WB for the confirmation of HTLV-1 and -2 infection (Campos et al., 2020; Okuma et al., 2020), but in cases of LIA- or WB-indeterminate and untyped results, molecular assays that amplify segments of the proviral DNA present in the HTLV-infected cells samples, must be conducted (Caterino-de-Araujo et al., 2021; Rosadas et al., 2021a). These molecular tests are based on polymerase chain reaction (PCR) in various formats: nested PCR (n-PCR), PCR followed by restriction fragment polymorphism analysis (PCR-RFLP), real-time quantitative PCR (qPCR), and loop-mediated isothermal amplification (LAMP) (Gomes et al., 2020; Caterino-de-Araujo et al., 2021; Rosadas et al., 2021a).

The HTLV Brazilian Protocol for Sexually Transmitted Infections 2020 recommends performing WB or LIA as the first confirmatory assay, and in case of inconclusive results conducting a PCR for confirmation (Rosadas et al., 2021a). However, at present, neither molecular protocols nor commercial molecular assays for HTLV-1/-2 diagnosis are commercially available in Brazil or validated by the Brazilian Ministry of Health regulatory agency (ANVISA). The molecular tests used in Brazil have been developed in-house and most of them have not been validated. This makes the implementation of routine molecular testing and the comparison of results from different laboratories difficult.

Since 1992, the Instituto Adolfo Lutz (IAL) Central Public Health Laboratory in São Paulo, Brazil, has been conducting studies on HTLV-1 and -2 infections, and has performed HTLV serology routinely since 1998. At this laboratory, we searched for the best assays and algorithms that could be used for testing in São Paulo and Brazil in general, based on performance and cost-effectiveness. During these years we had the opportunity to use WB (several trademarks and versions), LIA, n-PCR (*env*, *tax*, LTR), PCR-RFLP (*env*, *tax*), and qPCR (*pol*) as confirmatory assays (de-Araujo et al., 1994; Caterino-de-Araujo et al., 1998, 2014, 2015; Jacob et al., 2007, 2008; Morimoto et al., 2007; Costa et al., 2011; Campos et al., 2017a,b, 2020), and it was concluded that laboratories without appropriate infrastructure for molecular biology studies could confirm infection using LIA instead of WB (Campos et al., 2020). For laboratories that had the appropriate infrastructure and supplies to perform both the confirmatory tests (serological and molecular), molecular tests should be first-line test used, considering the better cost/benefit ratio (Caterino-de-Araujo et al., 2021). Of all the molecular confirmatory assays, qPCR has the advantage of quantifying the HTLV-1/2 proviral load (PVL), and consequently, stratifying the risk of HTLV-1-associated disease development (Haziot et al., 2019; Rosadas et al., 2021a).

A study of the literature published regarding the molecular assays for the diagnosis of HTLV-1/2, particularly in Brazil, revealed the differences in PCR protocols among laboratories, regions of the proviral DNA amplification, primer pairs and probes, PCR platforms and reagents, and criteria of positivity (Caterino-de-Araujo and Gonçalves, 2021).

In light of this scenario, and considering the exorbitant expenses on reagents, the shortage of clinical specimens, the different qPCR platforms, and the supplies available in each Central Public Health Laboratory in Brazil, we decided to develop and validate two in-house multiplex quantitative real-time PCR assays (mqPCR_HTLV), targeting the *pol* and *tax* genes, for the simultaneous identification of HTLV-1, HTLV-2, and the human albumin reference gene using double plasmids (pHTLV1-Alb and pHTLV2-Alb) for the standard curve construction. We used two platforms and seven commercial master mix formulations simulating the various core facility laboratories in Brazil and their different conditions and supplies.

## Materials and Methods

### Samples

Blood samples obtained from patients tested for HTLV-1/-2 specific antibodies which were infected and not infected with HIV-1, HBV and HCV were distributed in the following groups: Group 1, 37 patients infected with HTLV-1 alone confirmed by WB; Group 2, 152 patients infected with HIV-1 and reactive of HTLV-1/2 infection by screening assays; Group 3, 30 patients not infected with HTLV but infected with HIV-1 and/or HBV and/or HCV.

Briefly, samples of Group 1 belonged to blood donors from Recife, Pernambuco (Northeast Brazil), tested positive for HTLV-1 during the years 2012–2017, and sent to IAL for HTLV-1 subtype characterization (LTR, *env* and *tax* sequencing) using DNA extracted from peripheral blood mononuclear cells (PBMCs) isolated by Ficoll-Paque Plus density gradient centrifugation (GE Healthcare, Uppsala, Sweden). The HTLV-1 sequences from Recife were deposited in GenBank (Accession Numbers LTR: MF178246.1 to MF178269.1; *tax*: KY928553.1 to KY928577.1; *env*: KY928459.1 to KY928480.1), and the remaining DNA samples were tested for mqPCR_HTLV assays.

Samples from Group 2 and Group 3 belonged to patients infected with HIV/AIDS attending AIDS Reference Centers in São Paulo, and were sent to IAL during the years 2015–2018 (*n* = 2,991) for HTLV-1/2 diagnosis. The samples were used to detect the prevalence of HTLV-1 and HTLV-2 in HIV-1 infected patients from São Paulo, for HTLV-1/2 subtype surveillance, and for searching markers of diagnostic and prognostic values in retroviruses coinfection. The data of such research were published elsewhere (Caterino-de-Araujo et al., 2015; Campos et al., 2017a,b, 2020). Blood samples were collected in tubes containing EDTA, and the plasma and peripheral blood leukocytes (PBLs) were separated by centrifugation at 800 g for 10 min. Plasma samples were first tested for HTLV-1/2 specific antibodies using two immunoenzymatic assays (Gold ELISA HTLV I+II, REM Industria e Comercio Ltda, São Paulo, Brazil and Murex HTLV I+II, Diasorin, United Kingdom), and subsequently by WB (HTLV Blot 2.4, MP Biomedicals, Asia Pacific Pte. Ltd.), which is considered the reference method for confirming and differentiating between HTLV-1 from HTLV-2 infections in a majority of laboratories in Brazil and elsewhere. The PBL were stored at −20^°^C for further DNA extraction and qualitative qPCR analysis.

In some of the HTLV-1/-2-infected patients (Group 2) in clinical and laboratory follow-up tests (*n* = 16), PBMCs were isolated by Ficoll-Paque Plus density gradient centrifugation (GE Healthcare, Uppsala, Sweden), washed twice in phosphate buffered saline, and 10^6^ cells pellets stored at −20^°^C for further qPCR quantitative analysis.

All samples refer to the routine diagnosis and projects on HTLV-1/2 approved by the Ethics Committee for Research of IAL (Ministry of Health protocol numbers CAAE #55837316.0.0000.0059 and #52493316.1.0000.0059).

### DNA Extraction

Genomic DNA was extracted and purified from PBL and PBMC samples using Roche MagNA Pure^®^ LC Robot Instrument, (Mannheim, Germany), with the LC MagNA Pure Nucleic Acid Isolation kit I from Roche Diagnostics, (Mannheim, Germany), according to the manufacturer’s instructions, and eluted with 100 μL of PCR-grade water. The extracted DNA was aliquoted into 4 vials for subsequent analysis and thawed only once.

### Primers and Probes

Table 1 presents the sequences of primers and probes used in the mqPCR_HTLV (*pol* and *tax*) assays for HTLV-1 and HTLV-2 detection, their position in the genomic prototypes (ATK HTLV-1 infected cell line; GenBank accession number J02029.1), MoT (HTLV-2 infected cell line; GenBank accession number M10060.1), human albumin (GenBank accession number M12523.1), amplicon size, and the authors’ reference. The oligonucleotide concentrations were optimized using different concentrations of primers and probes in the range of 0.2–0.9 and 0.1–0.3 μM, respectively.

**TABLE 1:** Primers and probes employed in two multiplex qPCR_HTLV assays (*pol* and *tax*) for detecting HTLV-1 and HTLV-2.

Gene	Primer/probe	Sequences (5′→3′)	Nucleotide position^a^	Amplicon size (bp)	References
***pol***	HTLV-1 F	GAACGCTCTAATGGCATTCTTAAAACC	4,788–4,814		Tamegão-Lopes et al., 2006
	HTLV-1 R	GTGGTTGATTGTCCATAGGGCTAT	4,895–4,872	108	
	HTLV-1 Pb^b^	FAM- ACTTTACTGACAAACCCGACCTACCCATGG-BHQ1	4,828–4,857		
	HTLV-2 F	CAACCCCACCAGCTCAGG	4,740–4,757		
	HTLV-2 R	GGGAAGGTTAGGACAGTCTAGTAGATA	4,830–4,804	91	
	HTLV-2 Pb^b^	Cy5- TGGTCGAGAGAACCAATGGT**R**TAATCAAAA-BHQ2	4,760–4,789		
***tax***	HTLV-1 F	CGGATACCCAGTCTACGTGTT	7,359–7,379		
	HTLV-1 R	CAGTAGGGCGTGACGATGTA	7,458–7,439	100	Takenouchi et al., 2011
	HTLV-1 Pb	FAM-CTGTGTACAAGGCGACTGGTGCC-BHQ1	7,386–7,408		
	HTLV-2 F	CGATTGTGTACAGGCCGATTG	7,272–7,292		
	HTLV-2 R	CAGGAGGGCATGTCGATGTAG	7,347–7,327	76	Waters et al., 2011
	HTLV-2 Pb	Cy5-TGTCCCGTCTCAGGTGGTCTATGTTCCA-BHQ2	7,294–7,321		
	Albumin F	GCTCAACTCCCTATTGCTATCACA	16,222–16,245		Tamegão-Lopes et al., 2006
***Albumin***	Albumin R	GGGCATGACAGGTTTTGCAATATTA	16,351–16,327	130	
	Albumin Pb^b^	HEX-TCTCTTGTGGGCTGTAATCATCGTCTAGGC-BHQ1	16,290–16,319		

*F, Forward; R, Reverse; Pb, Probe; FAM, 6-carboxy-fluorescein; CY5, cyanine dye; HEX, phosphoramidite dye; BHQ, black hole quencher, R, degenerate base—recognition of A or G bases (in bold and underlined).*

*^a^Primer and probe nucleotide positions aligned with ATK (HTLV-1 infected cell line; GenBank accession number J02029.1), MoT (HTLV-2 infected cell line; GenBank accession number M10060.1) and human albumin, intron 12 (GenBank accession number M12523.1).*

*^b^Probes adapted/modified from Tamegão-Lopes et al. (2006).*

Comparative analysis of sequences of HTLV-1 and HTLV-2 isolates from Brazil and abroad using the Basic Local Alignment Search Tool of the National Center for Biotechnology Information (NCBI) website,^1^ showed a mismatch (A and G) among some HTLV-2 Brazilian isolates at position 4,780 of the *pol* target region. Thus, to overcome this problem, a degenerate base (R) was included in the probe design (see Table 1, in bold and underlined).

### Plasmids

Double recombinants plasmids of HTLV-1- and HTLV-2-albumin for both the gene targets (*pol* and *tax*) were employed as positive controls for standard curves construction.

The double recombinant plasmid of the *pol* target gene of HTLV-1 and the human albumin (pHTLV1pol-Alb) was kindly provided by Jorge Casseb from the Instituto de Medicina Tropical, São Paulo, and it was constructed by cloning a segment of 246 bp of the *po*l region of HTLV-1 (nucleotide position range from 4,708 to 4,953) and a 1,183 bp segment (nucleotide position range from 15,758 to 16,940) of intron 12 of the human albumin gene, according to Dehée et al. (2002). The pHTLV2pol-Alb double plasmid was kindly provided and designed by Marina Lobato Martins from the Fundação HEMOMINAS, Belo Horizonte, Minas Gerais, and consisted of a synthetic DNA of 321 bp, cloned into the vector pENO8H, containing a 138 bp fragment of the *pol* gene of HTLV-2 (nucleotide position range from 4,723 to 4,860) and a 170 bp fragment of the human albumin gene (nucleotide position range from 16,202 to 16,371).

The double recombinant plasmids for the *tax* target genes were designed at IAL and synthesized by Invitrogen GeneArt Gene Synthesis (Thermo Scientific, Waltham, United States). The pHTLV1tax-Alb plasmid consisted of a 321 bp synthetic DNA sequence (nucleotide position range from 7,339 to 7,488), and the pHTLV2tax-Alb plasmid consisted of a 297 bp fragment (nucleotide position 7,252–7,377), and both the plasmids contained a 170 bp fragment of the human albumin gene (nucleotide position range from 16,202 to 16,371). The plasmids containing the HTLV-1 or HTLV-2 *tax* and albumin target genes were expanded in *E. coli* cells in Luria Bertani (LB) medium containing 0.2 mg/mL ampicillin, and subsequently extracted and purified using the QIAprep Spin up Miniprep kit (Qiagen, Hilden, Germany). The sequences of the cloned inserts were determined and confirmed by performing a sequencing reaction on ABI 3130xl equipment and subsequently compared with reference sequences in GenBank (Accession Numbers J02029.1, M12523.1, and M10060.1).

The plasmid concentrations (ng/μL) were determined using the NanoDrop 2000 spectrophotometer (Thermo Fisher Scientific, Waltham, United States), and the Avogadro’s constant was used to convert it to the number of copies per microliter.

To determine the performance of the plasmids in mqPCR_HTLV assays, their standard curves and the limit of detection (LOD) were obtained by amplification of 10-fold dilutions of the plasmids containing 10^6^ to 1 copy/5 μL in Tris-EDTA solution (10 mM Tris, 1 mM EDTA) and pH 8.0, in duplicate. The cycle quantification (Cq) values obtained were plotted against the logarithm of copy number.

Since HTLV-2 is known to have fewer copy numbers in relation to HTLV-1 (Lee et al., 2004; Murphy et al., 2004; Montanheiro et al., 2008), the LOD for HTLV-2 (*pol*) assay with concentrations of plasmids varying from 100 to 10 copies was tested in six replicates among the range of 100–15 copies and in 12 replicates for 10 copies.

The efficiencies of the assays were calculated according to the formula E = 10^(–1/*slope*)^ −1 (Kubista et al., 2006; Raymaekers et al., 2009); the parameters for a singleplex assay were: efficiency 90–110% (slope between −3.6 and −3.1) and *R*^2^ ≥ 0.99; those for a multiplex assay were: efficiency 80–120% (slope between –3.9 and –2.9) and *R*^2^ ≥ 0.98 (Broeders et al., 2014).

### Standardization of Multiplex Quantitative Real-Time PCR for HTLV-1/-2 Assays

The serial dilution of plasmids were utilized in mqPCR_HTLV using the Roche LightCycler 480II (Roche Diagnostics, Indianapolis, Indiana) and the 7.500 Applied Biosystems—ABI (Applied Biosystems) platforms. Each mqPCR_HTLV reaction was 25 μL in volume and contained: 5 μL of DNA template, 12.5 μL of master mix reagent, 0.2 μM of each set of oligonucleotides, except for the *tax* probe, which was used at 0.1 μM. Passive reference dye (ROX) was added to the reaction mix when using the ABI platform, according to the manufacturer’s instructions. PCR thermocycler conditions for amplification were as follows: 1 cycle at 50^°^C for 2 min, 1 cycle at 95^°^C for 10 min, 50 cycles at 90^°^C for 50 s, and 1 cycle at 60^°^C for 1 min (Tamegão-Lopes et al., 2006; Costa et al., 2011). The reactions were conducted in duplicate and considered positive if the amplification curves displayed the shape/slope and characteristics of an amplification reaction with a Cq value ≤ 40, according to the LOD of assays.

Seven different commercial master mix brands for hydrolysis probes were analyzed in the mqPCR_HTLV assays, using the ABI/Roche platforms: LightCycler Probes Master (Roche), Platinum^®^Quantitative PCR SuperMix-UDG (Invitrogen), GoTaq^®^Probe qPCR Master Mix (Promega), PerfeCTa qPCR ToughMix (Quantabio); Sso Advanced™ Universal Probes Supermix (Bio-Rad), Kapa Probe Fast qPCR Kit Master Mix (2X) Universal (Kapa Biosystems) and LuminoCt^®^ qPCR ReadyMix™ (Merck/Sigma).

### Validation of Multiplex Quantitative Real-Time PCR for HTLV-1/-2 Assays

To validate the multiplex qPCR format using plasmids, we compared the performance of multiplex and singleplex reactions in detecting HTLV-1 and HTLV-2 (*pol* and *tax*) at six points along the standard curves.

The LOD and efficiency of the mqPCR_HTLV assays were evaluated simultaneously on ABI and ROCHE platforms using serial dilutions of the double plasmids. The same LOD protocol was followed for conducting the reactions with different master mix formulations, as well as for analyzing the reproducibility of the assay by three different analysts and that of the robotic workstation for high-precision dilution setup of PCR-QIAgility (Qiagen). These professionals reproduced the reactions without previous knowledge of the mqPCR_HTLV results, having received only the initial concentration of the plasmids and the LOD protocol.

### Performance of Multiplex Quantitative Real-Time PCR for HTLV-1/-2 Assays in Diagnosis

For calculating the efficiency of analytical performance of mqPCR_HTLV assays (*pol* and *tax*), we employed genomic DNA samples obtained from patients who were HTLV seropositive in screening assays, and for calculating the sensitivity and specificity of assays we considered the WB results. In case of divergent results, we observed the results of another confirmatory serological assay (LIA, INNOLIA HTLV-I/II, Fujirebio, Europe N.V., Belgium) and/or the PCR-RFLP (*tax*) assay, as previously described (Tuke et al., 1992; Campos et al., 2017b).

The sensitivity of the assays was evaluated in DNA samples obtained from PBL or PBMC of 37 patients infected with HTLV-1 alone (Group 1) and 152 patients infected with HIV and suspected of HTLV-1/-2 coinfection (Group 2). The specificity of assays was evaluated using genomic DNA samples of 30 HTLV-1/-2-seronegative patients infected with HIV-1 (Group 3), which included 22 HIV-monoinfected, 2 HIV/HBV-coinfected, 3 HIV/HCV-coinfected, and 3 HIV/HBV/HCV-coinfected.

The precision of mqPCR_HTLV (*pol* and *tax*) assays (reproducibility intra- and inter-assays) was assessed in DNA samples of patients that had different positive Cq values (profiles): Cq ≤ 30 (high); Cq from 31 to 35 (median); Cq from 36 to 37 (low); Cq from 38 to 40 (scarce). Eight clinical DNA samples were processed (4 HTLV-1 and 4 HTLV-2) in duplicate and the experiments were conducted daily across 4 consecutive days.

In addition to the qualitative use of the assays, we evaluated their possible use in PVL quantification. Unfortunately, due to the low volume of the remaining DNA samples, this analysis could not be performed for all the mqPCR_HTLV assays, but only mqPCR_HTLV (*pol*), which was conducted in duplicate using the ABI equipment. Using 5 μL of the 100 μL of DNA samples extracted from 1 × 10^6^ PBMCs of 16 HTLV-1/2-infected patients in follow-up (Group 2), we inferred that the Cq value obtained corresponds to 0.5 × 10^5^ cells. Thus, for calculating the number of copies of HTLV-1 and HTLV-2 in 10^5^ cells, we plotted the Cq value obtained in the standard curve and multiplied the number of copies by 2.

### Statistical Analysis

The mqPCR_HTLV assay data were analyzed by descriptive statistics using 2 × 2 contingency tables and the Microsoft Excel 2016 software. 95% confidence interval (CI) and the Kappa index were calculated. The Grubbs test was employed for the comparison of the methods and the creation of the linearity graphs.

The intra- and inter-assay reproducibility were calculated in percentage using the mean Cq, standard deviation (*SD*) values, and coefficient of variation (CV) (CV = 100 × standard deviation / mean, %). CV of Cq values of intra- and inter-assay reproducibility of 3 and 10%, respectively, are considered acceptable according to Dehée et al. (2002), and Estes and Sevall (2003).

## Results

### Multiplex Quantitative Real-Time PCR for HTLV-1/-2 (*pol* and *tax*) Assays Optimization

After testing several concentrations of primers (0.2–0.9 μM), and probes (0.1–0.3 μM), the mqPCR_HTLV (*pol* and *tax*) assays were optimized using 0.2 μM primers and probes, except for the *tax* probe which performed better at a concentration of 0.1 μM.

### Comparative Efficiency of Singleplex and Multiplex qPCR_HTLV Assays

**FIGURE 1:**
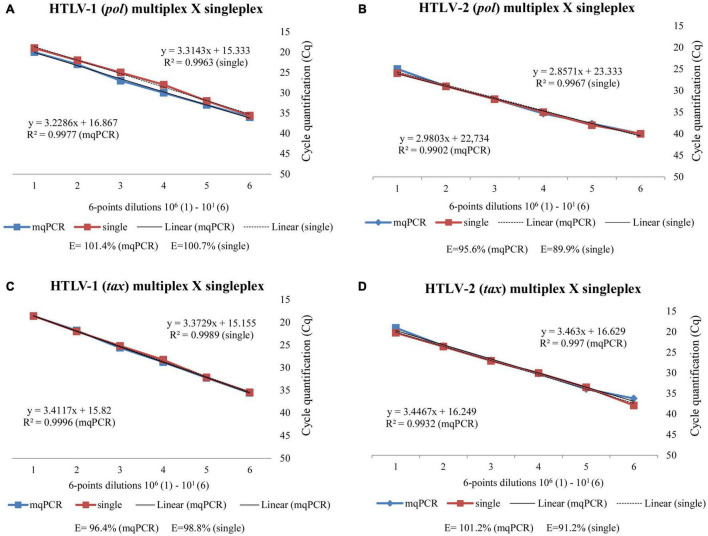
Comparative efficiency of the singleplex and multiplex qPCR_HTLV in detecting HTLV-1/-2 *pol* **(A,B)** and *tax* **(C,D)** target genes using serial plasmid dilutions and linear regression analyses, conducted in ROCHE platform.

Figure 1 represents the comparative efficiency of singleplex and multiplex qPCR_HTLV in detecting HTLV-1/-2 *pol* (Figures 1A,B) and *tax* (Figures 1C,D) target genes using serial plasmid dilutions and linear regression graphics analyses. Six synthetic DNA concentrations were detected in both the assays, with *R*^2^ values > 0.99 or 0.98 and at least 90 or 80% efficiency as acceptable values for the two detection assays, respectively.

### Efficiency and Limit of Detection of the Multiplex Quantitative Real-Time PCR for HTLV-1/-2 Assays With Respect to Plasmids Dilutions and Different Platforms

The efficiencies and LODs of mqPCR_HTLV (*pol* and *tax*) assays analyzed using the ABI and Roche’s equipment are shown in Table 2. Independent of the equipment utilized, the best LODs were set for detecting HTLV-1 and the *tax* target gene.

**TABLE 2:** Efficiency of the mqPCR_HTLV (*pol* and *tax*), slope and correlation coefficients (*R*^2^) for detecting HTLV-1 and HTLV-2 using two platform/equipment.

	gene	HTLV-1		HTLV-2	
Platforms		ABI	ROCHE^a^	ABI	ROCHE^a^
Efficiency %	*pol*	100.0	101.4	99.3	104.0
Slope		−3.32	−3.29	−3.33	−3.19
Linearity–*R*^2^		0.991		0.998	
LOD		10	10	70	70
Efficiency %	*tax*	110.6	100.2	96.2	100.2
Slope		−3.30	−3.31	−3.41	−3.31
Linearity–*R*^2^		0.996		1.000	
LOD		10	10	10	10

*LOD, limit of detection (copy per reaction); ABI, Applied Biosystems 7.500-ABI platform; ROCHE, LightCycler 480 II equipment.*

*^a^R^2^ not calculated by this equipment.*

All results obtained (*pol*, *tax* and Albumin) agree with the acceptance performance parameters for mqPCR assays, but because the LOD for HTLV-2 *pol* ranged from 10 to 100 copies per reaction, it was decided to conduct additional evaluations in this range of plasmid dilutions to determine the best final LOD using intra-assay reproducibility analyses (Table 3). The reactions were conducted in 6 replicates for concentrations ranging between 15 to 100 plasmid copy number, and in 12 replicates for 10 plasmid copy number. The results obtained disclosed 100% consistent detections (6/6) for 70 copies, 66% (4/6) for 50 copies, and 50% (3/6) for 20 copies (Table 3). Thus, we decided to consider 70 copies as the LOD for HTLV-2 (*pol*).

**TABLE 3:** Limit of detection and intra-assay reproducibility of the mqPCR_HTLV (*pol*) for detecting HTLV-2.

Copy number	positive + / negative – (HTLV-2_*pol*)	Reproducibility (%) (number of positive/number of replicates)
100	++++++	100 (6/6)
90	++++++	100 (6/6)
80	++++++	100 (6/6)
70	++++++	100 (6/6)
60	−/++++/−	66 (4/6)
50	++/−−/++	66 (4/6)
40	−−−−/++	33 (2/6)
30	−−/++/−−	33 (2/6)
20	−+++/−−	50 (3/6)
15	−−−−/+/−	16 (1/6)
10	−−−−−−/+/−/+/−−−	16 (2/12)

*Reactions conducted using plasmid number dilutions ranging from 100 to 10 copies, Kapa Biosystems master mix, ROCHE LightCycler 480II equipment, and six and twelve replicates.*

### Sensitivity and Efficiency of the Multiplex Quantitative Real-Time PCR for HTLV-1/-2 Assays Using Several Master Mix Formulations

**FIGURE 2:**
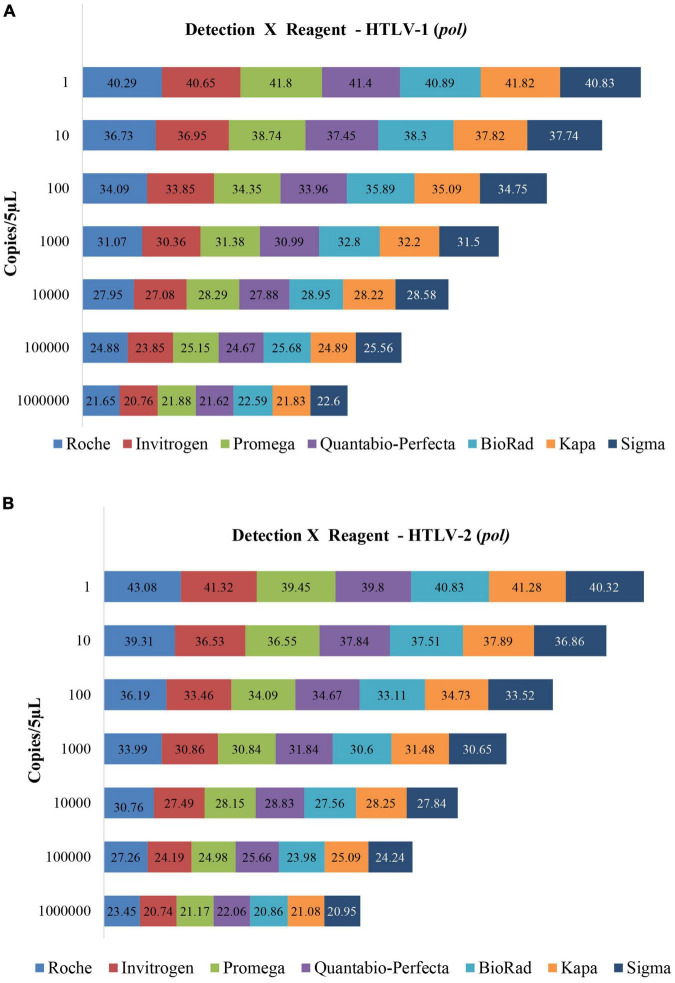
Limit of copy number detection of the mqPCR_HTLV assay (*pol*) for detecting HTLV-1 **(A)** and HTLV-2 **(B)** using plasmids and seven different master mix trademarks, and ROCHE LightCycler 480II equipment. The Cq values are disclosed inside the rectangles.

**FIGURE 3:**
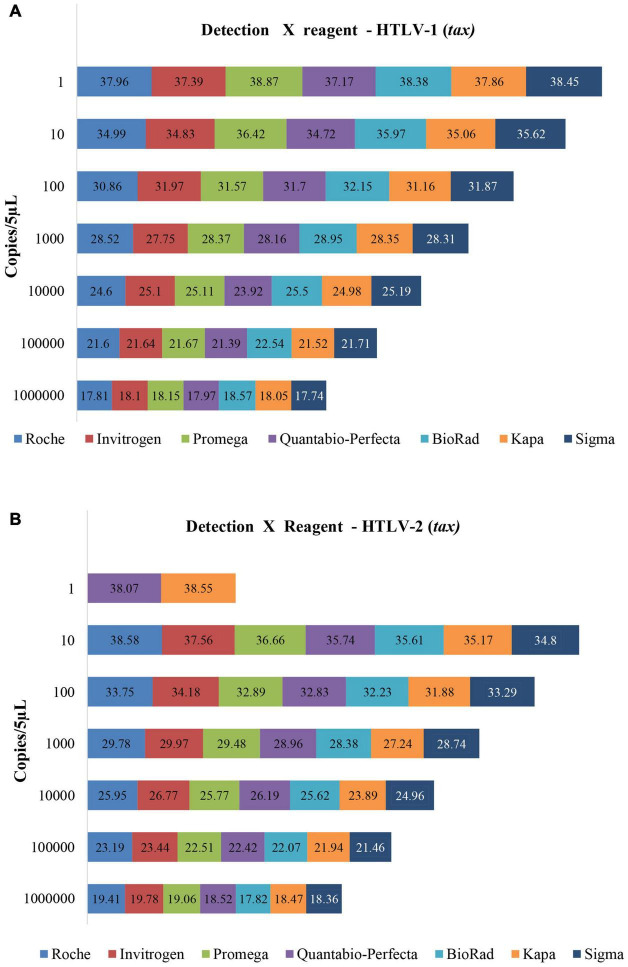
Limit of copy number detection of the mqPCR_HTLV assay (*tax*) for detecting HTLV-1 **(A)** and HTLV-2 **(B)** using plasmids and seven different master mix trademarks, and ABI 7.500 equipment. The Cq values are disclosed inside the rectangles.

We assessed the feasibility and performance of seven different commercial master mix reagents, available in the Brazilian market, for mqPCR assays for HTLV-1 and HTLV-2 provirus detection using the LOD protocol for *pol* and *tax* target genes. The results obtained are shown in Figures 2, 3. All commercial reagents exhibited the parameters required of the mqPCR assays, displaying good performance in the reactions, with the lowest efficiency being over 90% (Table 4), confirming the flexibility of use of master mix formulations in these assays.

**TABLE 4:** Efficiency values of the mqPCR_HTLV assays for detecting HTLV-1 and HTLV-2 (*pol* and *tax*) and the reference gene (human albumin gene), using seven commercial master mix reagents.

Efficiency of Master Mix solutions detections in the mqPCR_HTLV								
Trademark^a^	*pol* target gene				*tax* target gene			
	HTLV-1	RG	HTLV-2	RG	HTLV-1	RG	HTLV-2	RG
ROCHE	101.20%	111.70%	100.45%	97.80%	108.98%	94.20%	94.60%	99.40%
Invitrogen	100.30%	106.70%	100.45%	100.10%	102.90%	102.60%	91.24%	93.90%
Promega	98.20%	108.10%	107.80%	103.40%	105.15%	106.80%	94.60%	94.80%
Quanta	100.35%	100.35%	100.25%	108.00%	101.80%	99.60%	101.60%	93.20%
BioRad	100.45%	95.90%	100.35%	100.50%	100.10%	102.30%	93.37%	94.90%
Kapa	100.45%	107.40%	100.25%	100.10%	102.06%	107.60%	96.37%	94.20%
Sigma	100.35%	109.60%	102.00%	102.50%	105.90%	107.80%	94.17%	94.80%
Eff. Mean	100.14%	105.68%	101.65%	101.77%	103.84%	102.99%	95.14%	95.03%

*^a^Master mix names described in Materials and Methods. mqPCR assays conducted using plasmids in the ROCHE-LightCycler 480II (pol) and ABI 7.500 (tax) equipments. RG, Reference Gene (Albumin-target); Eff, efficiency.*

### Precision of Multiplex Quantitative Real-Time PCR for HTLV-1/-2 Assays

The intra- and inter-assay reproducibility results of the mqPCR_HTLV (*pol* and *tax*) assays conducted with respect to the plasmid dilutions prepared by the three analysts, and those prepared by the robotic workstation for high-precision PCR, are presented in Table 5. The data analysis showed no substantial differences among the professionals in reproducing the assays, with acceptable values of CV for all. All of them were able to detect at least 10 copies of HTLV-1 in both the mqPCR_HTLV (*pol* and *tax*) assays, 10 copies of HTLV-2 in mqPCR_HTLV (*tax*) assay, and approximately 10–100 copies of HTLV-2 in mqPCR_HTLV (*pol*) assay, thereby corroborating previous results.

**TABLE 5:** Intra- and inter-assay reproducibility of mqPCR_HTLV (*pol* and *tax*) for detecting HTLV-1 and HTLV-2 using serial plasmid dilutions prepared by three analysts and a robot, conducted in duplicate using the ABI equipment.

Target gene	Copies/5 μL	mqPCR_HTLV-1							mqPCR_HTLV-2						
		*QIAgility Cq	Analyst 1 Cq	Analyst 2 Cq	Analyst 3 Cq	Mean Cq	*SD*	CV%	*QIAgility Cq	Analyst 1 Cq	Analyst 2 Cq	Analyst 3 Cq	Mean Cq	*SD*	CV%
***pol***	1,000,000	20/20	20/20	21/21	21/21	20.5	0.5	2.6	23/23	23/23	24/24	25/25	23.8	0.9	3.7
	100,000	23/24	24/24	25/25	24/24	24.1	0.6	2.7	27/27	26/27	27/27	29/28	27.3	0.9	3.3
	10,000	27/27	27/27	28/28	27/27	27.3	0.5	1.7	30/30	30/30	31/31	32/32	30.8	0.9	2.9
	1,000	30/30	30/30	31/31	31/31	30.5	0.5	1.8	34/34	33/33	34/35	35/35	34.1	0.8	2.4
	100	34/33	34/34	35/35	34/34	34.1	0.6	1.9	37/37	36/37	37/38	37/38	37.1	0.6	1.7
	10	38/36	37/38	38/38	38/37	37.4	0.7	2.0	40/40	0/39	38/39	39/38	39.0	0.8	2.1
	1	0/37	0/39	0/0	38/0				0/0	0/0	0/0	0/0			
***tax***	1,000,000	18/18	18/19	17/18	18/19	18.1	0.6	3.5	19/20	20/21	20/19	20/19	19.8	0.7	3.6
	100,000	22/22	22/22	21/20	22/21	21.5	0.8	3.5	23/23	23/23	23/22	22/23	22.8	0.5	2.0
	10,000	25/25	26/26	24/24	25/25	25.0	0.8	3.0	26/26	25/26	27/25	26/26	25.9	0.6	2.5
	1,000	28/28	29/29	27/28	28/28	28.1	0.6	2.3	30/30	29/29	30/29	28/29	29.3	0.7	2.4
	100	31/31	32/32	30/31	30/31	31.8	0.8	2.2	33/33	31/31	33/33	33/33	32.5	0.9	2.8
	10	35/35	36/36	34/35	34/35	35.0	0.8	2.2	36/36	34/35	36/36	36/35	35.5	0.8	2.1
	1	38/39	0/0	45/0	38/37				39/38	0/39	0/0	38/0			

*QIAgility, robotic PCR setup; SD, standard deviation; CV, coefficient of variation (calculated between non-zero values).

### Performance of the Multiplex Quantitative Real-Time PCR for HTLV-1/-2 Assays in Diagnosis (Sensitivity and Specificity)

To calculate the sensitivity of the mqPCR_HTLV (*pol* and *tax)* assays we compared the WB results. Of the 37 DNA samples from patients infected with HTLV-1 alone analyzed by WB (Group 1), 36 were positive in both mqPCR_HTLV (*pol* and *tax*) assays (97.3%; CI 95% 76.9–84.6). The single mqPCR_HTLV negative sample showed discordant results on screening (data not shown).

The testing of 152 DNA samples from HIV-infected patients that were HTLV reactive on screening (Group 2) using the mqPCR_HTLV *pol* and *tax* assays showed different and interesting results (Table 6). Per WB results, 118 out of the 152 samples were HTLV positive (77.6%, 95% CI 76.9–84.6): 77 HTLV-1, 29 HTLV-2, 3 HTLV-1+2, 9 HTLV untyped, 22 indeterminate, and 12 negative. Per the mqPCR_HTLV (*pol*) assay, 101 out of the 152 samples were positive (66.4%, 95% CI 57–75.4), and as per the mqPCR_HTLV (*tax*) assay, 95 out of the 152 samples were positive (62.5%, 95% CI 52.7–72.2). The mqPCR_HTLV (*pol*) assay was able to detect more cases of HTLV-2 than the mqPCR_HTLV (*tax*) assay and WB (Table 6).

**TABLE 6:** Results of HTLV-1/2 confirmatory tests (WB, mqPCR_HTLV *pol* and *tax*) when employed in HIV-infected individuals suspected of HTLV-1/2 infection by screening assays (Group 2).

Confirmatory test	HTLV-1	HTLV-2	HTLV-1 and HTLV-2	HTLV	Ind	Neg	Pos	Total	Positivity %
WB	77	29	3	9	22	12	118	152	77.6
mqPCR_HTLV (*pol*)	69	31	1			51	101	152	66.4
mqPCR_HTLV (*tax*)	68	25	2*			57	95	152	62.5

WB, western blot; mqPCR_HTLV (pol), multiplex quantitative real-time PCR for pol target gene; mqPCR_HTLV (tax), multiplex quantitative real-time PCR for tax target gene; HTLV, HTLV untyped sample; Ind, indeterminate; Neg, negative; Pos, positive. *Three samples with discrepant results described in the text.

Among the 22 WB-indeterminate samples, the mqPCR_HTLV (*pol*) assay identified ten as positive (45.5%): nine as HTLV-2 (Patients N. 14, 20, 29, 35, 83, 116,144, 146, and 148), and one as HTLV-1 (Patient N. 7). By the mqPCR (*tax*) assay, seven were positive (31.8%): six were HTLV-2 (Patient N. 14, 29, 35, 83, 144, and 146) and one was HTLV-1 (Patient N. 7). Regarding the nine WB HTLV untyped samples, six (66.7%) were identified as positive: three HTLV-1 (Patient N. 31, 88, and 120) and one HTLV-2 (Patient N. 84), using both the mqPCR (*pol* and *tax*) assays, one HTLV-2 using mqPCR *pol* (Patient N. 129) and one HTLV-1 by mqPCR *tax* (Patient N. 108) (Table 7).

**TABLE 7:** Final results of HTLV-1/2 confirmatory tests (mqPCR_HTLV *pol* and *tax*, WB when employed in 152 HIV-infected individuals suspected of HTLV-1/2 infection by screening assays (Group 2).

N	mqPCR_*pol*	mqPCR_*tax*	WB	N	mqPCR_*pol*	mqPCR_*tax*	WB	N	mqPCR_*pol*	mqPCR_*tax*	WB
**1**	HTLV-1	NEG	HTLV-1	**26**	HTLV-1	HTLV-1	HTLV-1	**51**	HTLV-2	HTLV-2	HTLV-2
**2**	HTLV-1	HTLV-1	HTLV-1	**27**	NEG	NEG	HTLV-1	**52**	HTLV-2	HTLV-2	HTLV-2
**3**	HTLV-1/-2	HTLV-1/-2	HTLV-1/-2	**28**	HTLV-2	HTLV-2	HTLV-2	**53**	HTLV-1	HTLV-1	HTLV-1
**4**	HTLV-1	HTLV-1	HTLV-1	**29**	HTLV-2	HTLV-2	IND	**54**	HTLV-1	HTLV-1	HTLV-1
**5**	HTLV-2	HTLV-2	HTLV-2	**30**	NEG	NEG	IND	**55**	NEG	NEG	HTLV-2
**6**	HTLV-1	HTLV-1	HTLV-1	**31**	HTLV-1	HTLV-1	HTLV	**56**	HTLV-1	HTLV-1	HTLV-1/-2*
**7**	HTLV-1	HTLV-1	IND	**32**	HTLV-1	HTLV-1	HTLV-1	**57**	HTLV-1	HTLV-1	HTLV-1
**8**	HTLV-1	HTLV-1	HTLV-1	**33**	HTLV-1	HTLV-1	HTLV-1	**58**	HTLV-1	HTLV-1	HTLV-1
**9**	NEG	NEG	HTLV-1	**34**	HTLV-1	HTLV-1	HTLV-1	**59**	HTLV-1	HTLV-1	HTLV-1
**10**	HTLV-2	HTLV-2	HTLV-2	**35**	HTLV-2	HTLV-2	IND	**60**	HTLV-1	HTLV-1	HTLV-1
**11**	HTLV-1	HTLV-1	HTLV-1/-2*	**36**	HTLV-2	HTLV-2	HTLV-2	**61**	HTLV-1	HTLV-1	HTLV-1
**12**	HTLV-1	HTLV-1	HTLV-1	**37**	HTLV-1	HTLV-1	HTLV-1	**62**	NEG	NEG	IND
**13**	HTLV-1	HTLV-1	HTLV-1	**38**	HTLV-1	HTLV-1	HTLV-1	**63**	HTLV-1	HTLV-1	HTLV-1
**14**	HTLV-2	HTLV-2	IND	**39**	HTLV-2	NEG	HTLV-2	**64**	HTLV-1	HTLV-1	HTLV-1
**15**	HTLV-1	HTLV-1	HTLV-1	**40**	HTLV-2	HTLV-1/-2*	HTLV-2	**65**	HTLV-1	HTLV-1	HTLV-1
**16**	NEG	NEG	NEG	**41**	HTLV-2	HTLV-2	HTLV-2	**66**	HTLV-1	HTLV-1	HTLV-1
**17**	HTLV-1	HTLV-1	HTLV-1	**42**	HTLV-1	HTLV-1	HTLV-1	**67**	HTLV-1	HTLV-1	HTLV-1
**18**	HTLV-1	HTLV-1	HTLV-1	**43**	HTLV-1	HTLV-1	HTLV-1	**68**	HTLV-1	HTLV-1	HTLV-1
**19**	NEG	NEG	NEG	**44**	HTLV-1	HTLV-1	HTLV-1	**69**	HTLV-1	HTLV-1	HTLV-1
**20**	HTLV-2	NEG	IND	**45**	HTLV-1	HTLV-1	HTLV-1	**70**	HTLV-1	HTLV-1	HTLV-1
**21**	NEG	NEG	HTLV-1	**46**	HTLV-2	HTLV-2	HTLV-2	**71**	HTLV-1	HTLV-1	HTLV-1
**22**	NEG	NEG	NEG	**47**	NEG	NEG	NEG	**72**	NEG	NEG	HTLV-2
**23**	NEG	NEG	HTLV-1	**48**	HTLV-2	HTLV-2	HTLV-2	**73**	NEG	NEG	IND
**24**	HTLV-2	HTLV-2	HTLV-2	**49**	NEG	NEG	HTLV-2	**74**	HTLV-1	HTLV-1	HTLV-1
**25**	HTLV-2	HTLV-2	HTLV-2	**50**	HTLV-1	HTLV-1	HTLV-1	**75**	NEG	NEG	NEG
**76**	HTLV-1	HTLV-1	HTLV-1	**102**	NEG	NEG	IND	**128**	HTLV-1	NEG	HTLV-1
**77**	NEG	NEG	HTLV-1	**103**	HTLV-1	HTLV-1	HTLV-1	**129**	HTLV-2	NEG	HTLV
**78**	NEG	NEG	IND	**104**	HTLV-1	HTLV-1	HTLV-1	**130**	NEG	NEG	HTLV-1
**79**	HTLV-2	HTLV-2	HTLV-2	**105**	NEG	NEG	HTLV-2	**131**	HTLV-1	HTLV-1	HTLV-1
**80**	HTLV-1	HTLV-1	HTLV-1	**106**	NEG	NEG	HTLV	**132**	NEG	NEG	IND
**81**	NEG	NEG	NEG	**107**	HTLV-1	HTLV-1	HTLV-1	**133**	HTLV-1	HTLV-1	HTLV-1
**82**	NEG	NEG	NEG	**108**	NEG	HTLV-1	HTLV	**134**	HTLV-1	HTLV-1	HTLV-1
**83**	HTLV-2	HTLV-2	IND	**109**	NEG	NEG	HTLV-2	**135**	HTLV-1	HTLV-1	HTLV-1
**84**	HTLV-2	HTLV-2	HTLV	**110**	NEG	HTLV-1	HTLV-1	**136**	HTLV-1	HTLV-1	HTLV-1
**85**	NEG	NEG	NEG	**111**	NEG	NEG	IND	**137**	HTLV-1	HTLV-1	HTLV-1
**86**	HTLV-2	HTLV-2	HTLV-2	**112**	NEG	NEG	HTLV-2	**138**	NEG	HTLV-1	HTLV-1
**87**	NEG	NEG	HTLV-1	**113**	HTLV-1	HTLV-1	HTLV-1	**139**	HTLV-1	HTLV-1	HTLV-1
**88**	HTLV-1	HTLV-1	HTLV	**114**	HTLV-1	HTLV-1	HTLV-1	**140**	HTLV-1	NEG	HTLV-1
**89**	NEG	NEG	HTLV-1	**115**	NEG	NEG	HTLV-1	**141**	NEG	NEG	IND
**90**	HTLV-1	HTLV-1	HTLV-1	**116**	HTLV-2	NEG	IND	**142**	HTLV-2	HTLV-2	HTLV-2
**91**	NEG	NEG	NEG	**117**	HTLV-1	HTLV-1	HTLV-1	**143**	HTLV-1	HTLV-1	HTLV-1
**92**	HTLV-1	HTLV-1	HTLV-1	**118**	NEG	NEG	HTLV-2	**144**	HTLV-2	HTLV-2	IND
**93**	NEG	NEG	NEG	**119**	HTLV-1	NEG	HTLV-1	**145**	HTLV-2	HTLV-2	HTLV-2
**94**	NEG	NEG	NEG	**120**	HTLV-1	HTLV-1	HTLV	**146**	HTLV-2	HTLV-2	IND
**95**	NEG	NEG	HTLV-1	**121**	HTLV-2	HTLV-2	HTLV-2	**147**	HTLV-2	HTLV-2	HTLV-2
**96**	NEG	NEG	NEG	**122**	NEG	NEG	HTLV	**148**	HTLV-2	NEG	IND
**97**	NEG	NEG	IND	**123**	NEG	NEG	IND	**149**	NEG	NEG	HTLV-1
**98**	HTLV-1	HTLV-1	HTLV-1	**124**	NEG	NEG	HTLV-1	**150**	NEG	NEG	HTLV-2
**99**	HTLV-1	HTLV-1	HTLV-1	**125**	NEG	NEG	IND	**151**	HTLV-1	HTLV-1	HTLV-1
**100**	NEG	NEG	IND	**126**	HTLV-1	HTLV-1	HTLV-1	**152**	HTLV-2	HTLV-2	HTLV-2
**101**	NEG	NEG	HTLV-2	**127**	NEG	NEG	HTLV				

*N, patient code number; mqPCR_HTLV (pol), multiplex quantitative real-time PCR for pol target gene; mqPCR_HTLV (tax), multiplex quantitative real-time PCR for tax target gene; WB, western blot (HTLV Blot 2.4, MP Biomedicals); HTLV, HTLV WB untyped sample; Ind, indeterminate; Neg, negative.*

*Discrepant results described in the text, HTLV-1 positive by PCR-RFLP (tax). Results according to in section “Materials and Methods”.

Furthermore, three discrepant results were observed among the confirmatory assays, one sample confirmed HTLV-2 infection in three tests (WB, LIA and mqPCR_HTLV *pol*), and was repeatedly detected positive for HTLV-1 and HTLV-2 by mqPCR-*tax* and for HTLV-1 by PCR-RFLP (*tax*) (Patient N. 40, Table 7), data published elsewhere (Campos et al., 2017b). The other two samples were confirmed HTLV-1 and HTLV-2 by WB analysis but were only HTLV-1 positive by mqPCR_HTLV (*pol* and *tax*) assays (Patients N. 11 and 56). In one of these samples the LIA was indeterminate.

Thus, the relative sensitivity of the mqPCR_HTLV (*pol* and *tax*) assays compared to that of WB in HTLV-1 monoinfected patients was 97.4%; in HIV/HTLV-coinfected was 77.1%, Kappa index—0.405 (95% CI 0.238–0.575) (mqPCR_HTLV *pol*) and 74.6%, Kappa index—0.435 (95% CI 0.277–0.593) (mqPCR_HTLV *tax*). No sample from Group 3 (HIV- and/or HBV- and/or HCV-infected) and or samples negative per WB and LIA assays from Group 2 were positive in mqPCR_HTLV assays. Additionally, all samples tested negative for HTLV-1 and HTLV-2 in molecular assays were positive for the reference gene (Albumin).

Table 8 shows the intra- and inter-assay reproducibility for detecting HTLV-1 and -2 using the mqPCR_HTLV assays (*pol* and *tax*). The DNA samples from eight individuals with different positive Cq profiles were tested in duplicates across four consecutive days. The mean Cq and CV values were determined and the results showed minor variation and good reproducibility of results in samples with low DNA concentrations (Cq > 36). Although the same input volume of DNA was used for both the assays to detect HTLV-2, a difference around 3 Cq was detected, the greater value being for *pol* target gene detection. Despite the fact that the mqPCR_HTLV *tax* presented lower Cq values in relation to the mqPCR_HTLV *pol*, the *tax* target gene was more difficult to detect in samples with scarce DNA copy number, corroborating previous findings of assays standardization.

**TABLE 8:** Intra- and inter-assay reproducibility of mqPCR_HTLV assays (*pol* and *tax*) for detecting HTLV-1 and HTLV-2 using DNA samples from eight individuals with different positive Cq profiles, in duplicate and in four consecutive days of analysis.

Positive profiles	HTLV-1+ (*pol*)							HTLV-1+ (*tax*)					HTLV-2+ (*pol*)							HTLV-2+ (*tax*)				
	S	REP	Cq	Mean	SD	CV%	ND	Cq	Mean	SD	CV%	ND	S	REP	Cq	Mean	SD	CV%	ND	Cq	Mean	SD	CV%	ND
High	**1**	1	28.9	29	0.4	1.5	8/8	28.8	29	0.1	0.5	8/8	**5**	1	32.2	33	0.2	0.5	8/8	29.7	30	0.4	1.2	8/8
			28.0					28.9							32.5					29.7				
		2	28.9					28.8						2	32.5					29.8				
			28.9					28.8							32.5					30.0				
		3	29.0					28.9						3	32.8					30.7				
			29.4					29.2							32.5					30.5				
		4	29.5					28.8						4	32.3					29.6				
			28.6					28.7							32.6					29.7				
Median	**2**	1	32.7	33	0.3	0.9	8/8	32.3	33	0.4	1.2	8/8	**6**	1	35.8	36	0.4	1.1	8/8	32.9	33	0.7	2.1	8/8
			32.2					33.3							35.4					32.8				
		2	32.6					32.2						2	35.8					32.9				
			32.6					33.3							35.7					32.7				
		3	32.6					32.8						3	35.9					34.2				
			32.4					32.5							36.2					32.8				
		4	32.6					32.6						4	35.8					32.9				
			33.4					32.4							34.7					31.3				
Low	**3**	1	35.8	36	0.2	0.6	8/8	35.8	36	0.6	1.8	8/8	**7**	1	39.3	39	0.5	1.3	8/8	34.9	36	1	2.8	8/8
			35.6					35.7							38.0					36.4				
		2	35.9					34.9						2	39.4					34.5				
			36.4					34.5							39.3					36.4				
		3	36.0					36.9						3	39.4					38.2				
			36.1					35.7							38.2					35.7				
		4	35.8					35.9						4	38.5					36.4				
			35.9					35.2							38.5					35.9				
Scarce	**4**	1	0.0	*38	18	46.7	2/8	*37.5	*38	18	46.7	2/8	**8**	1	0.0	*40	17	43.8	1/8	0.0	0	0	0.0	0/8
			0.0					*37.6							0.0					0.0				
		2	0.0					0.0						2	0.0					0.0				
			0.0					0.0							0.0					0.0				
		3	*37.0					0.0						3	0.0					0.0				
			0.0					0.0							0.0					0.0				
		4	0.0					0.0						4	0.0					0.0				
			*37.8					0.0							*40					0.0				

S, samples; REP, repetition/days; Cq, Cycle quantification/amplification value; SD, Standard deviation; CV, Coefficient of variation; ND, number of detections. *Calculated between non-zero values; Positive profiles: high (Cq < 30), median (Cq from 31 to 35), low (Cq from 36 to 37) and scarce (Cq from 38 to 40).

### Application of Multiplex Quantitative Real-Time PCR for HTLV-1/-2 Assays for Proviral Load Measurement

Sixteen DNA samples from patients of Group 2 in clinical and laboratory follow-ups were analyzed for PVL quantification using the mqPCR_HTLV (*pol*). Four (25%) were negative, seven (43.7%) were HTLV-1 positive, and five (31.3%) were HTLV-2 positive. The results of provirus copy number calculated as described in section “Materials and Methods” are presented as Supplementary Table 1. Additionally, all samples that were negative for HTLV-1 and HTLV-2 in mqPCR_HTLV assays had DNA of good quality and quantity, indicated by the positive results of the albumin reference target gene.

## Discussion

Molecular confirmatory assays, mostly qPCR, are important tools for HTLV-1/2 diagnosis, for avoiding vertical transmission in children born to HTLV-1/2-infected mothers, and for monitoring HTLV-1/2 PVLs and disease development (Rosadas et al., 2021b). Serological confirmatory assays for HTLV-1 and HTLV-2 (WB and LIA) are expensive and prone to delivering inconclusive results (indeterminate or HTLV untyped results) (Campos et al., 2020; Caterino-de-Araujo et al., 2021). In Brazil, all molecular assays for HTLV diagnosis are developed in-house but only a few of them were validated (Andrade et al., 2010; Waters et al., 2011; Furtado et al., 2012; Ribeiro et al., 2012; Rosadas et al., 2013; Caterino-de-Araujo and Gonçalves, 2021). The aim of the present study was to develop and validate two multiplex qPCR_HTLV assays capable of simultaneously detecting the presence of HTLV-1 and HTLV-2 target genes and the albumin reference gene, using different PCR platforms and master mix formulations. This design and validation aimed to solve problems related to time constraints, cost, low quantity of biological specimens, and the diversity of PCR platforms and reagents available in the Public Health Laboratories in Brazil.

We used four double synthetic plasmids for standard curve construction (two of which were customized by us), since this kind of reference material is stable, provides reproducible results, and could be used for absolute PVL quantification (Dehée et al., 2002; Kamihira et al., 2010; Abad-Fernández et al., 2014; Bandeira et al., 2020). Bandeira et al. (2020) identified the potential of using synthetic oligonucleotides to prepare a standard curve for HTLV-1 PVL quantification based on the linearity, qPCR efficiency, and equivalent results of such references when compared with a validated qPCR assay using a conventional TARL-2 cell standard curve, as constructed by Rosadas et al. (2013). In addition, these authors highlighted that procuring and maintaining such reference material is less expensive than using DNA obtained from cells lineages infected with HTLV-1 and -2 (Bandeira et al., 2020).

We selected two segments of the proviral genome of HTLV-1 and -2 to standardize these assays, *pol*, and *tax*, and utilized probes labeled with FAM (HTLV-1), CY5 (HTLV-2) and HEX (albumin). We used two assays employing different target genes to avert possible false negative results due to the presence of point mutations in primers and probes binding regions or the presence of defective provirus particles (absence of such genes) in HTLV-1 and -2 infected individuals (Tamiya et al., 1996; Ramirez et al., 2003; Takenouchi et al., 2011; Kuramitsu et al., 2017; Campos and Caterino-de-Araujo, 2020; Blanco et al., 2021; Caterino-de-Araujo and Campos, 2021). We evaluated theses assays in two Platforms (ROCHE and ABI) and with seven master mix formulations.

Comparative performance analysis of the plasmid standard curves construction and sensitivities in singleplex and multiplex assays showed no difference, which indicates that there is no interference of one labeled probe with another labeled probe in the multiplex qPCR_HTLV assays. The comparative analyses of the standard curves showed similar efficiency, and the overall sensitivities (LOD) of assays were 10 and 10 copies for HTLV-1 and 10 and 70 copies for HTLV-2 using *tax* and *pol* target genes, respectively, with efficiencies above 90%.

Although previous studies of mqPCRs disclosed greater sensitivities for detect and quantify HTLV-1/2 compared to the present study, they are restricted to equipment and reagents, with trademark exclusivity. For instance, Moens et al. (2009) developed a triplex qPCR for simultaneous detections of 1 copy of HTLV-1 (FAM), HTLV-2 (JOE), and HTLV-3 (CY5) using *tax* specific target primers and human β-globin as reference gene (NED). However, the reference gene to quantify cells number was detected separately and the dye NED is exclusive to the ABI platform.

Lee et al. (2004) developed one biplex qPCR assay using a primer pair that amplifies a segment of *tax* gene common to HTLV-1 and HTLV-2, and as reference gene another single qPCR for HLA-DQ alpha; both assays employed the Sybr Green dye. Thus, although these qPCR showed high sensitivity (1 copy per reaction), and could be employed in different PCR platforms, the biplex qPCR does not distinguish HTLV-1 from HTLV-2. Estes and Sevall (2003) described another biplex qPCR that utilizes the *tax* segment of HTLV-1 and a segment of the 5′ LTR of HTLV-2 as targets and the singleplex assay for human β-globin for PVL quantification. In these assays, the ABI equipment and supplies were utilized and a LOD of ∼60 copies were detected for both HTLV-1 and HTLV-2.

Besson and Kazanji (2009) developed a fourplex qPCR to detect HTLV-1, HTLV-2, HTLV-3, and human albumin. The authors used primer pairs for the *pX* regions of HTLV with LOD of 1 copy for HTLV-1 and 10 copies for HTLV-2. Nevertheless, the probe used for HTLV-1 *pX* detection was labeled with ROX. Since this dye is a passive reference that the ABI platform needs to normalize the fluorescence data, this component does not allow the use of such fourplex qPCR for quantitative analyses in this equipment, as well as commercial reagents that have ROX or similar passive reference in their formulation.

Waters et al. (2011) developed and validated a triplex qPCR for detecting the segments *pol* of HTLV-1, *tax* of HTLV-2, and the human albumin as reference. They used the labeled probes HTLV-1 (JOE), HTLV-2 (FAM) and Albumin (NED) and detected sensitivities varying from 1 to 10 copies for all target genes but solely using the ABI platform. Thus, considering the previous information, the present mqPCR_HTLV assays have the advantage of being used in two different platforms, representative of the PCR platforms available in Public Health Laboratories in Brazil.

Furthermore, the present mqPCR_HTLV showed similar sensitivity (LOD) using seven different master mix formulations, solving the problem of using varied brands of master mix depending on availability and cost.

The concern reported around the LOD (more sensitivity) in the previous qPCR assays compared with the present mqPCR_HTLV assays, is that the discordant results could be partially explained by the target choice. When we used the *pol* as target gene, differences in sensitivity (LOD) of assays were detected. In contrast, when we used the *tax* target gene a significant LOD was detected, corroborating with previous descriptions. The majority of mqPCR assays above employed the *tax* gene for HTLV-1/2 detection. We do not know the cause of these results, but despite the lower LOD of the mqPCR *pol*, this assay was able to detect 1 copy of HTLV-1 and 10 copies of HTLV-2, but without consistent reproducibility. In this regard, according to the Poisson’s distribution, LOD < 3 copies, and the detections of 1 copy of DNA are considered only hypothetical to qPCR assays (Bustin et al., 2009), and needed to define the dynamic range of the method and the PCR characteristics (Raymaekers et al., 2009). Detection below the LOD IC 95% could be obtained in DNA samples from pathogens, but with less accuracy (Kralik and Ricchi, 2017), corroborating the present results. Although the LOD = 1 copy is possible when conducted usually with plasmids, in clinical samples they will be incredibly rare, except for digital-PCR.

Curiously, although Cq values > 40 were obtained in mqPCR_HTLV (*pol*) assay using plasmids, when this assay was employed in clinical DNA samples, no sample showed Cq values > 40. Thus, we could suggest in the future, after gaining experience from testing many samples, to reduce the number of cycles repetition from 50 to 45, and consider the Cq value ≤ 40 for positive samples.

Another important fact that emerged from this study was the greater diagnostic sensitivity of mqPCR_HTLV (*pol*) for detecting samples infected with HTLV-2, which seems contradictory with the fact that this assay has a lower LOD compared to mqPCR_HTLV (*tax*). HTLV-2 is more difficult to be confirmed by serological and molecular confirmatory assays, as HTLV-2 accounts for more indeterminate WB results (Morimoto et al., 2007; Costa et al., 2011; Caterino-de-Araujo et al., 2014, 2015; Campos et al., 2017b,^2020^) and has a low PVL (Lee et al., 2004; Murphy et al., 2004; Montanheiro et al., 2008).

The relative sensitivity of 97.4% detected in samples from patients monoinfected with HTLV-1 in both mqPCR assays of the present study is in accordance with a series of studies reported in a review of molecular assays for HTLV-1/2 diagnosis (Caterino-de-Araujo and Gonçalves, 2021); the unique sample that resulted negative on these assays presented discordant results in serologic screening, which could be due to low PVL (under the detection limit of assays) and consequently low levels of antibodies production, and/or the presence of provirus mutations or defective particles circulating in this patient.

Among samples of HIV/HTLV-coinfected patients, relative sensitivities of 77.1% (mqPCR-HTLV *pol*) and 74.6% (mqPCR_HTLV *tax*) were detected and the negative results could be due to: (i) provirus mutations and defective HTLV particles circulating in HIV/HTLV-coinfected patients (Campos and Caterino-de-Araujo, 2020; Caterino-de-Araujo and Campos, 2021), (ii) fluctuations in HTLV-1/-2 PVL in patients under antiretroviral therapy (Machuca and Soriano, 2000), (iii) use of highly active antiretroviral therapy (HAART) for long periods of time by the patients (Costa et al., 2011; Caterino-de-Araujo et al., 2015), and (iv) low PVL, mostly of HTLV-2 (Lee et al., 2004; Murphy et al., 2004; Montanheiro et al., 2008). Despite these limitations, the present mqPCR_HTLV were able to confirm and distinguish HTLV-1 and HTLV-2 infections in HIV-infected patients that resulted WB-indeterminate or HTLV untyped, as occurred in the past using the singleplex qPCR assays (Costa et al., 2011; Caterino-de-Araujo et al., 2015). As expected, no complete agreement of results was detected between mqPCR assays and WB (Kappa index = 0.4), since they are designed for different targets (proviral DNA and antibodies, respectively).

Notably, both mqPCR_HTLV assays disclosed 100% specificity when applied in samples of patients infected with HIV, and/or HBV, and/or HCV. In patients with HIV infection, the correct diagnosis of HTLV-1 and HTLV-2 coinfection has predictive value, since they impact in HIV disease progression to AIDS: HTLV-1 accelerates HIV disease progression while HTLV-2 has been pointed to delay progression to AIDS (Beilke, 2012).

Concerning the use of present assays for PVL quantification, although only the mqPCR_HTLV (*pol*) could be evaluated, the results obtained support its use for monitoring infection and stratifying the risk for HTLV-1-associated disease development (Haziot et al., 2019; Rosadas et al., 2021a).

Finally, although the present study had some limitations regarding: (i) limited volume of biological samples to conduct all comparative experiments, (ii) absence of samples of patients infected only with HTLV-2, (iii) possible low PVL in HIV-infected patients on HAART treatment, (iv) insufficient volume of samples for validate PVL quantification for both target genes (*pol* and *tax*), and (v) no inter-labs comparative analysis, the present mqPCR_HTLV assays were validated for routine diagnosis considering the parameters previously described for qualitative assays (standard curves, efficiencies, *R*^2^ values), without false positive results.

In conclusion, the present mqPCR_HTLV (*pol* and *tax*) assays displayed feasibility in diagnosing HTLV-1 and HTLV-2 in different core facilities laboratory, conditions, and supplies.

## Data Availability Statement

The original contributions presented in the study are included in the article/Supplementary Material, further inquiries can be directed to the corresponding author/s.

## Ethics Statement

The studies involving human participants were reviewed and approved by Research Ethics Committee of Instituto Adolfo Lutz. The patients/participants provided their written informed consent to participate in this study.

## Author Contributions

MG designed the work, performed all the experiments, figures and tables, analyzed the results, and wrote the manuscript. LF designed and expanded the plasmids, conducted some experiments, analyzed the results, and wrote the manuscript. FH conducted the experiments of reproducibility. KC conducted experiments that supported to determine the diagnostic performance of the new assays. ACA conceived and administrated the project, acquired funding, supervised the experiments, analyzed the results, and wrote the manuscript. All authors read and approved the submitted version.

## Conflict of Interest

The authors declare that the research was conducted in the absence of any commercial or financial relationships that could be construed as a potential conflict of interest.

## Publisher’s Note

All claims expressed in this article are solely those of the authors and do not necessarily represent those of their affiliated organizations, or those of the publisher, the editors and the reviewers. Any product that may be evaluated in this article, or claim that may be made by its manufacturer, is not guaranteed or endorsed by the publisher.

## Footnotes

1
http://www.ncbi.nlm.nih.gov/BLAST/
