# Supplementary material for: Development and Validation of Multiplex Quantitative Real-Time PCR Assays for Simultaneous Detection and Differentiation of HTLV-1 and HTLV-2, Using Different PCR Platforms and Reagent Brands
Source: Front Microbiol. 2022 Mar 15;13:831594. doi: 10.3389/fmicb.2022.831594 (PMC8965094; doi:10.3389/fmicb.2022.831594)
Supplement: Supplementary file 1 [file Table_1.pdf]

Table 1. Proviral load quantification in peripheral blood mononuclear cells samples of seven HIV/HTLV-1-coinfected and five HIV/HTLV-2-coinfected patients on clinical and laboratory follow-up, using the mqPCR_HTLV (*pol*) assay and plasmids as reference material.

**Sample code**	**HTLV-1 Copy number**			**HTLV-2 Copy number**			**Reference gene**
	**Cq**	**[5uL]**	**[10^5 cells]**	**Cq**	**[5uL]**	**[10^5 cells]**	**Cq**
**1**	0	0	0	27	136,000	272,000	24
**2**	32	245	490	0	0	0	23
**3**	27	9,380	18,760	0	0	0	24
**4**	0	0	0	0	0	0	24
**5**	0	0	0	36	260	520	23
**6**	0	0	0	0	0	0	23
**7**	0	0	0	33	2,020	4,040	24
**8**	27	9,140	18,280	0	0	0	23
**9**	31	633	1,266	0	0	0	23
**10**	0	0	0	0	0	0	25
**11**	0	0	0	0	0	0	23
**12**	0	0	0	33	2,390	4,780	23
**13**	33	135	270	0	0	0	25
**14**	26	21,100	42,200	0	0	0	24
**15**	0	0	0	33	1,960	3,920	23
**16**	31	953	1906	0	0	0	23

The copy number in 10^5 cells was extrapolated from the copy number obtained per reaction (5 µL) as described in Material and Methods section.
